# Supplementary material for: Collaborative intelligence in AI: Evaluating the performance of a council of AIs on the USMLE
Source: PLOS Digit Health. 2025 Oct 9;4(10):e0000787. doi: 10.1371/journal.pdig.0000787 (PMC12510544; doi:10.1371/journal.pdig.0000787)
Supplement: S2 File — (ZIP) [file pdig.0000787.s002.zip › S2 Data Raw data files containing unprocessed question inputs and LLM outputs including transcripts of deliberations/STEP3_Question-121-1_Type-MC-NJ.docx]

**Facilitator** **[Round 1]:** A 70-year-old man, who was admitted to the hospital 3 days ago for treatment of pneumonia, has now developed diarrhea and severe lower abdominal cramping. The patient has passed watery and yellow stools every 2 hours for the past 12 hours. Since admission, the patient has been treated with intravenous levofloxacin therapy and albuterol/ipratropium nebulizers every 4 hours, with only mild improvement of his respiratory symptoms. Medical history is also significant for hypertension, type 2 diabetes mellitus, and hyperlipidemia. Routine medications include rosiglitazone, amlodipine, and atorvastatin. He has smoked one pack of cigarettes daily for the past 50 years. BMI is 20 kg/m2. Vital signs are temperature 36.7°C (98.0°F), pulse 102/min, respirations 16/min, and blood pressure 100/50 mm Hg. Pulse oximetry on room air shows an oxygen saturation of 93%. Physical examination discloses dry mucous membranes and cracked lips. Lungs are clear to auscultation bilaterally. Cardiac examination discloses a tachycardic but regular rhythm. Abdomen is diffusely tender to palpation with no distention, rebound, or guarding. Test of the stool for occult blood is negative. Results of serum laboratory studies are shown: Urea nitrogen - 30 mg/dL Creatinine - 1.5 mg/dL Calcium - 8.2 mg/dL Na⁺ - 150 mEq/L K⁺ - 3.7 mEq/L Cl⁻ - 115 mEq/L HCO3⁻ - 22 mEq/L Glucose - 120 mg/dL Stool culture is obtained. Intravenous infusion of which of the following substances is the most appropriate next step? (A) Bicarbonate (B) Calcium gluconate (C) 5% Dextrose in water (D) 0.9% Saline

**AI_C:** RESTATING THE QUESTION: So, the scenario describes a 70-year-old individual with several comorbidities, who was admitted to the hospital because of pneumonia. This patient has now developed new lower abdominal pain and progressively worsening diarrhea, likely as an adverse reaction to his medical treatments. These are significant symptoms that require appropriate clinical management to alleviate discomfort and prevent further complications. Laboratory results point toward dehydration, reflected in high urea levels and sodium concentration, probably due to excessive loss of fluids through diarrhea. In this context, the question asks about the most appropriate intravenous substance to be administered to this patient. PLANNING: The first assumption is that the administration of a certain intravenous substance can improve his condition. Several possibilities are given, including bicarbonate, calcium gluconate, 5% dextrose in water, and 0.9% saline. Each of these options has different implications for the patient's physiology. Bicarbonate is typically used as a buffer in conditions of acidosis, but the patient's bicarbonate level is in the normal range. Calcium gluconate is a calcium supplement, but the patient's calcium level is adequate. Dextrose provides an energy source but doesn't address the main problem, which is fluid and electrolyte loss. Lastly, 0.9% saline is an isotonic solution commonly used for volume expansion and electrolyte replacement, but without additional nutrients or energy sources. CLINICAL REASONING: The patient is experiencing diarrhea, a condition often accompanied by significant loss of fluids and electrolytes, such as sodium and chloride. This can lead to a state of dehydration, which seems to be the case here, evidenced by the patient's elevated urea and sodium levels, and his physical manifestations of dry membranes and cracked lips. Hydration status has significant implications for several bodily functions, including kidney function and cardiovascular responsiveness. Dehydration can lead to renal impairment and circulatory collapse if not treated properly. CONCLUDING: Thus, addressing the patient's state of dehydration is vital. The priority should be to replenish his body with fluids and electrolytes that have been lost through diarrhea. Given the patient's elevated sodium level, a solution that can provide both water and the appropriate balance of electrolytes is ideal. RESPONSE: Therefore, the most appropriate intravenous substance to administer in this situation is (D) 0.9% Saline. It is an isotonic solution that would provide volume expansion and electrolyte replenishment, addressing the patient's immediate needs.

**AI_B:** RESTATING THE QUESTION: This is a scenario about a 70-year-old man who initially came to the hospital for pneumonia but has developed diarrhea and lower abdominal cramps now. He's been treated with levofloxacin and albuterol/ipratropium nebulizers for pneumonia. Medical history reveals hypertension, type 2 diabetes mellitus, and hyperlipidemia, and he is on rosiglitazone, amlodipine, and atorvastatin. He has been a smoker for the past 50 years. A recent physical examination showed symptoms of dehydration such as dry mucous membranes, and cracked lips. The abdominal examination revealed diffuse tenderness. Laboratory reports are also given. The question asks for the most appropriate next intravenous substance to be given to the patient under such clinical conditions. PLANNING: Initial assumptions in this scenario could be dehydration due to diarrhea, misbalance of electrolytes, and a potential infection due to hospitalization and antibiotic use. An alternate assumption to consider is antibiotic-associated diarrhea, specifically Clostridioides difficile infection, which is typically associated with antibiotic use in hospitalized patients. Given the symptoms of diarrhea, lower abdominal pain, and the recent use of antibiotics in the clinical scenario, this assumption should be clinically prioritized. CLINICAL REASONING: The frequent watery stools, lower abdomen cramping, and patient's exposure to healthcare environment and antibiotics especially levofloxacin point towards the likelihood of a C. difficile infection. This bacteria by producing toxins, causes symptoms like watery diarrhea, abdominal pain and could lead to severe complications like pseudomembranous colitis. Hypertension, diabetes mellitus type 2 and hyperlipemia won't directly cause diarrhea, but these comorbidities require careful monitoring and accurate treatment for fluid and electrolyte balance. The history of smoking for 50 years which might have led to chronic obstructive pulmonary disease (COPD), which could explain the initial presentation of pneumonia and the use of albuterol/ipratropium nebulizer. From the laboratory reports, we can see the patient has hypernatremia (elevated sodium levels), suggestive of dehydration, which is common in patients with prolonged diarrhea. The stool culture will help to confirm the C. difficile infection. CONCLUDING: Given the entire clinical context, diarrhea, lower abdominal cramping, dehydration, and antibiotic use, C. difficile infection is a strong possibility. Therefore, the immediate medical management should aim at correcting the dehydration (caused by the diarrhea) and hypernatremia. RESPONSE: The most appropriate step in this case would be to give an intravenous infusion of 0.9% Saline (D), which will help in rehydration and in correcting the hypernatremic state, the most critically altering lab value at this moment.

**AI_A:** (1) RESTATING THE QUESTION: This 70-year-old male patient was hospitalized for pneumonia, but has since started experiencing lower gut issues, including cramps and diarrhea occurring frequently. His antibiotic and inhaler treatment hasn't entirely curbed his respiratory issues. He has a history of hypertension, type 2 diabetes, and high cholesterol, and takes corresponding medication. He's a long-term smoker and has a BMI of 20 kg/m2. His vital signs are relatively stable, though he demonstrates signs of dehydration and tachycardia. His abdomen is sensitive but doesn't show serious signs of internal damage. Stool tests were negative for blood, and blood tests results are provided. The patient's stool has been cultured. What intravenous substance should he receive next? (2) PLANNING: A vital element to consider in this situation is the recent use of Levofloxacin, an antibiotic. Antibiotics may cause a Clostridium difficile infection, a bacterial infection that can cause diarrhea. Thus, we should operate under the strong assumption that the patient's gastrointestinal symptoms are likely due to antibiotic-driven clostridium difficile colitis. Also, his lab results indicate a high sodium level often associated with dehydration, which is backed up by the physical exam findings. This leads us to prioritize rehydration as an immediate step in treatment. (3) CLINICAL REASONING: The signs of dehydration including dry mouth, cracked lips, tachycardia, and a sodium level of 150 mEq/L (normal range is 135-145 mEq/L) seem to indicate intracellular dehydration due to loosing fluid in his gastrointestinal tract. Intense diarrhea as experienced by the patient can steal significant amounts of water and electrolytes from the body, and it's crucial to correct this imbalance quickly not only to ease symptoms but also to prevent further complications like kidney damage. (4) CONCLUDING: Given these parameters, it would be crucial to replenish lost fluids and electrolytes to correct the imbalance created by his possible clostridium difficile colitis. Rehydrating the patient should also help correct his sodium levels and may alleviate some of his tachycardia and physical signs of dehydration, which would improve circulatory and cognitive functions, among other things. (5) RESPONSE: In this situation, the most appropriate next step would be the intravenous infusion of 0.9% Saline (Option D). Saline solution mimics the body's extracellular fluid, providing both water and sodium to correct dehydration and electrolyte imbalances, which are likely causing tachycardia and high sodium levels in our patient. Other options may not address the current pressing issue of dehydration adequately.

**AI_E:** RESTATING THE QUESTION: This question pertains to a 70-year-old man with a history of hypertension, type 2 diabetes mellitus, and hyperlipidemia, who was admitted for treatment of pneumonia cumulatively three days ago, and has now developed diarrhea and severe abdominal cramps. He has been passing watery, yellow stools approximately every two hours for the past 12 hours. He has been managed with intravenous levofloxacin and albuterol/ipratropium nebulizers every 4 hours; however, this has led only to slight improvement in his respiratory symptoms. The patient's results from different laboratory studies need to be interpreted for clinical decision-making. The question is asking what the most appropriate next step in treatment is, specifically in terms of the intravenous infusion of a substance. PLANNING: The patient's clinical picture and recent history of being administered antibiotics (levofloxacin), showing new onset diarrhea and abdominal cramping, points toward the possibility of Clostridium difficile infection. This is particularly likely when antibiotic use alters the normal gut flora and allows pathogenic bacteria like C. difficile to flourish, leading to pseudomembranous colitis. Levofloxacin, a fluoroquinolone, is a high-risk antibiotic for C. difficile infection. The patient's laboratory findings show raised urea nitrogen level and creatinine level, possibly suggesting acute kidney injury, which could be related to dehydration subsequent to the diarrheal losses. The vital signs also indicate dehydration with tachycardia and low blood pressure. The high sodium level (hypernatremia) suggests that the patient may be suffering from relative water deficiency, another finding indicative of dehydration. CLINICAL REASONING: Given these findings, the patient should be treated with prompt fluid resuscitation to correct the volume deficit and stabilize his hemodynamics while stool test results are pending. Fluid choice for resuscitation depends on patient status and electrolyte abnormalities. Ideally, a hypotonic fluid like 0.45% saline or a balanced crystalloid (eg, lactated Ringer solution) would be appropriate given his hypernatremia. However, these options are not available in the choices given. Therefore, 0.9% saline (normal saline), an isotonic fluid, should be an appropriate choice under these circumstances. CONCLUDING: Based on the above lines of reasoning, prompt fluid resuscitation with 0.9% saline should be the priority in the patient's care. Fecal transplantation, antibiotic therapy, or surgical treatment may later be considered based on additional information, like the result of the stool culture or clinical course, to treat the potential C. difficile infection. RESPONSE: The most appropriate next step should be intravenous infusion with (D) 0.9% Saline.

**AI_D:** (1) RESTATING THE QUESTION: This elderly man has recently been hospitalized for pneumonia and has been on antibiotic therapy. Nevertheless, he developed diarrhea and abdominal cramping. We also know that he has persistent respiratory symptoms mildly improved with bronchodilator therapy, and has a significant medical history including hypertension, type 2 diabetes, and high cholesterol levels. His physical examination findings of dry mucous membranes and cracked lips could indicate dehydration. The fundamental answer we need is regarding the appropriate intravenous infusion for him given his case presentation and lab results. (2) PLANNING: The primary assumption is that the patient's diarrhea and abdomen cramping might be due to the antibiotic therapy (levofloxacin) he received for pneumonia. Many antibiotics can disrupt the normal gut microbiota leading to overgrowth and toxin production by pathogenic bacteria such as Clostridium difficile. An alternate assumption could be that he developed diarrhea due to another cause, but the recent antibiotic use pushes the assumption towards antibiotic-associated diarrhea. Hypernatremia and elevated creatinine may indicate dehydration. Around this, we must prioritize managing the fluid and electrolyte disturbances. (3) CLINICAL REASONING: Levofloxacin is a fluoroquinolone antibiotic associated with a risk of C. difficile-associated diarrhea. The watery, yellow stools and lower abdominal cramping suggest this possibility; therefore, a stool culture is appropriately obtained. However, the severe ongoing fluid losses from diarrhea have resulted in hypernatremia (150 mEq/L), likely due to dehydration as supported by physical signs such as dry mucous membranes, cracked lips, tachycardia (102/min), and slight hypotension (100/50 mm Hg). His BUN and creatinine are also mildly elevated, suggesting renal insufficiency due to hypovolemia and dehydration. The electrolyte imbalance and dehydration need urgent correction to maintain adequate organ perfusion. (4) CONCLUDING: Given the above clinical reasoning, the most suitable intervention would involve correcting his hypernatremia and dehydration. Calculated osmolarity shows a hypertonic state likely due to loss of a larger proportion of water than solutes, creating a "concentration" of remaining solutes in the serum. Correcting hypernatremia too quickly can lead to cerebral edema and neurologic damage; hence a solution isotonic to the patient's tonicity, but not contributing significantly to further elevating serum sodium, and containing water for rehydration would be preferred. (5) RESPONSE: Therefore, considering the patient's apparent hypernatremia and dehydration, the most appropriate next intravenous infusion would be (D) 0.9% Saline. This essentially provides water and sodium in equal parts, allowing for gradual correction of hypernatremia and dehydration.

**Facilitator** The consensus among the AI responses is that the 70-year-old male patient, who was initially hospitalized for pneumonia and has since developed diarrhea and abdominal cramping, is likely suffering from dehydration and electrolyte imbalance. This is potentially due to a Clostridium difficile infection, which could have been caused by the antibiotic Levofloxacin that he was administered for his pneumonia. The patient's lab results, including high sodium levels, and physical symptoms such as dry mucous membranes and cracked lips, further support this diagnosis. Given these factors, the AI responses agree that the most appropriate next step in treatment would be the intravenous infusion of 0.9% Saline. This isotonic solution would provide both water and sodium, helping to correct the patient's dehydration and electrolyte imbalances. Other options, such as bicarbonate, calcium gluconate, or 5% dextrose in water, are not deemed suitable as they do not adequately address the patient's immediate needs.
